# Intraoperative aortic endograft placement for an unexpected plaque rupture during lung surgery

**DOI:** 10.1016/j.ijscr.2019.06.001

**Published:** 2019-06-08

**Authors:** Wei Huang, Beatrice Aramini, Jiang Fan

**Affiliations:** a Department of Thoracic Surgery, Tongji University Shanghai Pulmonary Hospital, Postal address: No. 507 Zheng Ming Road, Shanghai 200433, PR China; b Department of Medical and Surgical Sciences for Children and Adults, University of Modena and Reggio Emilia, 41124 Modena, Italy

**Keywords:** CT, computed tomography, PET/CT^18^F-FDG, positron emission tomography with 2-deoxy-2-[fluorine-18]fluoro-d-glucose integrated with computed tomography, SUV, max standardized uptake value maximum, FEV1, forced expiratory volume in the 1st second, FVC, forced volume vital capacity, EBUS, endobronchial ultrasound, MAP, mean arterial pressure, ICU, intensive care unit, Aortic endograft, Aortic bleeding, Lung tumor invasion, Challenging procedure, Teamwork

## Abstract

**Background:**

Surgical resection of tumors invading the aorta is a challenging procedure. More recently, the use of thoracic aortic endografts has been reported to facilitate en bloc resection of tumors invading the aortic wall. The best treatment option is to keep the procedure separated before lung resection to reduce the risks of bleeding, therefore avoiding adverse consequences for the patient. However, an aortic stent placement before surgery is not mandatory with no clear signs of tumor or atherosclerotic plaque infiltrating the entire aortic wall.

**Case presentation:**

A 72-year-old man came to our Department for a persistent cough. Computed tomography (CT) scan with enhancement showed a mass located in the left upper lobe of the lung with no clear sign of infiltration or calcified plaques along the entire vascular wall. A positron emission tomography with 2-deoxy-2-[fluorine-18] fluoro-d-glucose integrated with computed tomography (PET/CT with 18F-FDG) was positive for hypermetabolic mass with negative lymph node stations bilaterally. Patient was undergone surgery for major lung resection by left thoracotomy. For an unexpected intraoperative bleeding due to the rupture of a calcified plaque, a stent was placed before proceeding with lung surgery. Patient was persistently stable, discharged after six days from surgery with no morbidities.

**Conclusions:**

In our case, no signs of the atherosclerotic plaque infiltration as well as no tumor infiltration were shown. In these situations, the aortic stent placement is possible in emergency, even during another operation. Nevertheless, surgeon experience and the good coordination among specialists is mandatory to yield a satisfying solution.

## Highlights

• Surgical resection of tumors invading the aorta is a challenging procedure.
• Thoracic aorta endografts facilitate *en bloc* resection of tumor infiltrating the aortic wall.
• The best treatment option is to keep the procedure separated before lung resection.
• Aortic stent placement is not mandatory with no clear signs of plaque infiltrating.
• The aortic stent placement is possible in emergency, even during another operation.

## 1 Introduction

Locally advanced lung cancer invading the aortic wall represents a major challenge for thoracic surgeons. Preoperative endovascular stent graft placement has been normally used to facilitate sectioning of tumors infiltrating the aorta [[1], [2], [3], [4]]. However, the presence of an aortic calcified plaque in the tumor area is not a “red flag” for possible bleeding during surgery, as well as a stent placement is not mandatory whenever a plaque is shown closed to the tumor, especially in the absence of clear signs of infiltration of the entire vascular wall. We describe a case of bleeding from injury of an adventitial plaque during mass mobilization before the lung tumor resection. Intraoperative positioning of an aortic endograft resolved the bleeding with the possibility to proceed with the operation [5]. The work has been reported in line with SCARE criteria has been reported in line with the SCARE criteria [6].

## 2 Case presentation

**Fig. 1:**
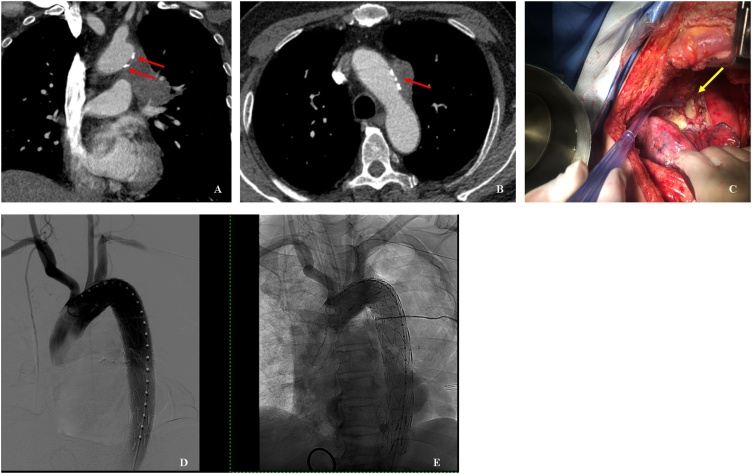
A and B. CT scan showing the left upper lobe tumor mass infiltrating the main left pulmonary artery and the bronchus. Fig. 1C. Red arrows highlighting the position of the atherosclerotic plaque. Fig. 1D and E. Revealing the endograft stent placed in the aorta by the interventional radiologist through the femoral artery. CT: computer tomography.

A 72-year-old man came to our Thoracic Surgery Department in March 2018 for a persistent cough that was resistant to therapy. He underwent chest X-ray and a CT scan with enhancement, which showed a mass of 34 × 32 mm located in the left upper lobe of the lung, infiltrating the left main pulmonary artery and the left bronchus (Fig. 1). No signs of an atherosclerotic plaque or tumor infiltration involving the entire aortic wall were detected. Invasion of a calcified plaque was slight and appeared to involve only the adventitia. A PET/CT with ^18^F-FDG was positive (Standard Uptake Value, SUV max = 15) for hypermetabolic mass with negative lymph node stations bilaterally. The patient had a smoking history (one pack/50 pack-years), with no other previous malignancies, and 20 years of comorbidities, including diabetes mellitus type 2, hypertension and hypercholesterolemia treated with medical therapy. Pulmonary function tests before surgery showed a forced expiratory volume in the 1st second (FEV1) of 1.7, equal to 80% predicted, and a forced volume vital capacity (FVC) of 2.4, equal to 82% predicted. The endobronchial ultrasound (EBUS) showed no N2 lymph node infiltration, although the percutaneous lung mass biopsy was highly suspect for adenocarcinoma of the lung. After signing the consent, the patient underwent a double-sleeve left upper lobectomy plus *en bloc* resection of the aortic wall adventitia for 4 × 2 cm^2^ through a left posterior thoracotomy. At the beginning of the operation, during the mobilization of the mass, a 5-mm aortic rupture occurred in the adventitia due to the presence of an atherosclerotic calcified plaque at this level (Fig. 1C). At first, manual pressure was applied on the bleeding site, then the surgeon tried to place a suture on the bleeding site, but the hardness of the plaque hindered the maneuvering. In the same time, the anesthesiologist maintained low mean arterial pressure (MAP). Patient conditions stayed persistently stable and after 15 min the bleeding was under controlled, however, for the high risk of an another unexpected bleeding, the thoracic wall was closed and the patient intubated was transferred urgently to the interventional operating room where an endovascular stent was placed from the left subclavian artery to the descending aorta using percutaneous retrograde common femoral artery access (Fig. 1D and E). After the vascular procedure, the patient was retransferred to the operative room, the chest was re-opened for inspection and for proceeding with the lung resection.

**Fig. 2:**
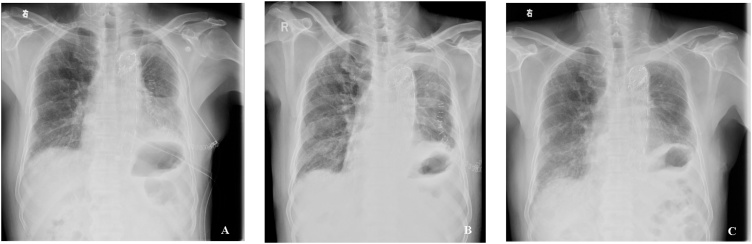
A. The chest X-ray on the day of discharge from the hospital (sixth postoperative day), at one month (2B) and four months after surgery (2C).

Intraoperative blood loss was totally 800 ml. Clinical parameters were stable during and after the procedure. The patient tolerated the endovascular stent placement and the subsequent double-sleeve left upper lobectomy within six total hours of operation. The patient was placed in the intensive care unit (ICU) for 48 h after surgery for optimal stabilization of his clinical condition. Chest tubes were removed on the third postoperative day, and the patient was discharged after six days from surgery with no morbidities (Fig. 2A). No stent-related complications were noted. Histology confirmed the diagnosis of adenocarcinoma of the lung, stage pT2 pN1 pM0 (TNM 8th), and an oncologic evaluation was requested for the next treatment options. Chest X-rays at one-month and four-month follow-up from surgery (Fig. 2B–C) revealed no complications.

## 3 Conclusions

The resection of the invaded adventitia of the aorta is a common procedure in thoracic surgery and the presence of atherosclerotic plaques is fairly common. In our case, after mobilization of the tumor mass, a calcified plaque lost the support inducing rupture of the adventitia of the aorta with consequent bleeding. In summary, even if it is possible to control and solve the aortic bleeding with an intraoperative stent placement and a good coordination among specialists, whenever it is possible to study and define accurately before the operation the characteristics of an atherosclerotic aortic plaque [5], especially the large ones, a stent should be placed prior to surgery.

## Conflicts of interest

The Authors have no financial and personal relationships to disclose.

## Sources of funding

No funding.

## Ethical approval

For single case report NO ethical approval needs. Patient signed a consent for publishing the case report.

## Consent

Patient signed a consent for the publication of this case report.

## Author contribution

W.H. and B.A. wrote the case report. J.F. revised the case report.

## Registration of research studies

Ethical Board approval is not required for case reports in our Center.

## Guarantor

Prof. Jiang Fan is the Guarantor of this case report.

## Provenance and peer review

Not commissioned, externally peer-reviewed.
